# Prior Psychiatric Disorder and Post-Traumatic Stress, Depressive and Anxiety Disorder after Traumatic Brain Injury with Glasgow Coma Scale Score 13–15: A TRACK-TBI Study

**DOI:** 10.1177/2689288X251383348

**Published:** 2025-09-30

**Authors:** Shawn R. Eagle, Lindsay D. Nelson, Joseph T. Giacino, Michael A. McCrea, Jason Barber, Nancy Temkin, John K. Yue, David O. Okonkwo, Geoffrey T. Manley, Murray B. Stein, the TRACK-TBI Investigators

**Affiliations:** ^1^ University of Pittsburgh, Pittsburgh, Pennsylvania, USA.; ^2^ Medical College of Wisconsin, Milwaukee, Wisconsin, USA.; ^3^ Harvard Medical School, Cambridge, Massachusetts, USA.; ^4^ University of Washington, Seattle, Washington, USA.; ^5^ University of California San Francisco, San Francisco, California, USA.; ^6^ University of California San Diego, La Jolla, California, USA.

**Keywords:** anxiety, depression, post-traumatic stress disorder, risk factor, traumatic brain injury

## Abstract

The most consistent risk factor for developing psychiatric problems post-traumatic brain injury (TBI) is a preexisting psychiatric disorder, but many studies have reported that psychiatric disorders can occur *de novo* following “mild” TBI. The objective of this secondary analysis of a prospective cohort study of patients (*n* = 1,947) with acute TBI and presenting Glasgow Coma Scale (GCS) score between 13 and 15 was to describe the association of pre-injury psychiatric history with prevalence and risk factors for probable posttraumatic stress disorder (PTSD), major depressive disorder (MDD), and nonspecific anxiety disorder (ANX) following TBI at long-term follow-up. Rates of probable PTSD, MDD, or ANX were analyzed from years 1 to 7 post-injury. Multivariable regression models were built to predict meeting cutoffs for PTSD, MDD, and ANX at 1 year, 2–4 years, and 5–7 years post-injury. Predictors were psychiatric history, migraine history, TBI history, initial head CT scan, loss of consciousness, post-traumatic amnesia, GCS, insurance type, highest level of care, cause of injury, years of education, age, sex, and race/ethnicity. Participants with history of pre-injury psychiatric disorder met clinical cutoffs for PTSD, MDD, and ANX at approximately double the rate of participants without history of psychiatric disorder at year 1 (16–28% vs. 5–13%), between years 2 and 4 (15–35% vs. 6–18%), and between years 5 and 7 (10–28% vs. 5–12%). Multivariable modeling confirmed several classical risk factors for post-TBI psychiatric sequelae at any time during follow-up, such as psychiatric history, prior TBI, female sex, and Black race. Assault, mechanism of injury, and Medicaid, self-pay, or other insurance (reference: private insurance) were also associated with PTSD, MDD, and ANX at various timepoints. Probable PTSD, MDD, and ANX were more common in participants with pre-injury psychiatric history, but significant psychiatric symptoms are identifiable for years post-injury, even among those with *de novo* psychiatric disorders.

## Introduction

Traumatic brain injury (TBI) with presenting Glasgow Coma Scale (GCS) score 13–15 represents approximately 80% of the TBIs seen in emergency departments (EDs) worldwide.^1^ Despite historically being considered a “mild” type of brain injury, longitudinal studies of TBI patients evaluated in EDs indicate that >50% have long-term functional limitations.^2^ psychiatric symptoms have been shown to precede functional limitations in this population, and the rate of long-term psychiatric symptoms and/or conditions ranges from 12% to 57% in the first year post-TBI.^3–5^ The most consistent risk factor for developing post-injury psychiatric problems is a preexisting psychiatric disorder, but many studies have reported that psychiatric disorders can occur *de novo* following “mild” TBI. In patients with moderate to severe TBI, prior studies have reported incident psychiatric disorder rates as high as 57% within the first 5 years of injury;^6^ however, few comparable long-term studies exist for adult “mild” TBI cohorts.

The primary aim of the present study was to evaluate the association of pre-injury psychiatric diagnosis with prevalence of probable (i.e., meeting or exceeding clinical cutoffs for validated screening tools) posttraumatic stress disorder (PTSD), major depressive disorder (MDD), and nonspecific anxiety disorder (ANX) following TBI with presenting GCS score 13–15 at 1–7 years post-injury. The secondary aim of this study was to use multivariable modeling to identify the most robust predictors of meeting or exceeding clinical cutoffs for these disorders at 1, 2–4, and/or 5–7 years post-TBI with GCS 13–15.

## Methods and Materials

### Design and participants

The Transforming Research and Clinical Knowledge in Traumatic Brain Injury (TRACK-TBI; ClinicalTrials.gov #NCT02119182) study enrolled 2,697 participants with TBI presenting to 1 of 18 United States (U.S.) Level 1 trauma center EDs through convenience sampling between February 26, 2014 and July 30, 2018, with final follow-up on June 30, 2019. For the current analysis, we restricted to those ≥17 years of age (excludes 145 participants), GCS 13–15 at presentation (excludes 552 with GCS <13), and those with unknown psychiatric history (excludes 52). The final analyzed sample included 1,948 participants. Participants or their legally authorized representatives provided written informed consent to participate after being approached by a member of the research team in the hospital. Human subjects research approvals were obtained by the institutional review board or ethics committee of each enrolling site, with the institutional review board of the University of California San Francisco serving as the ethics reviewer. This paper was structured within the Strengthening the Reporting of Observational Studies in Epidemiology guidelines.

Participants were included in the study if they presented to the hospital within 24 h of external force trauma to the head and met the American Congress of Rehabilitation Medicine’s criteria for diagnosis of TBI.^7^ The treating physician must also have ordered a head CT scan for the patient to be enrolled. Exclusion criteria included pregnancy, incarceration, nonsurvivable physical trauma, debilitating pre-injury mental health disorders (e.g., schizophrenia, bipolar disorder) or neurological disease (e.g., stroke, dementia), and non-English- or Spanish primary language. Ideally, outcome measures were collected in person for the 1-year visit, but if not possible, they were conducted over the phone. Outcome measures for timepoints >1 year were conducted over the phone.

### Outcome measures

#### Demographics and Injury Characteristics

A trained researcher collected relevant demographic, self-reported medical history (including history of a psychiatric diagnosis), and injury information including age, biological sex, race (white, Black, Asian, “Other” or unknown), Hispanic ethnicity (yes/no/unknown), years of education, injury cause (motor vehicle crash occupant, motor cycle collision [MCC], motor vehicle accident as a cyclist or pedestrian, fall, assault, other/unknown), hospital status (ED only, admitted to hospital, admitted to intensive care unit), GCS, loss of consciousness (yes/no), post-traumatic amnesia (yes/no), initial CT scan to detect acute intracranial abnormality (positive vs. negative),^8^ prior TBI history (none, ED only, admitted to hospital), and insurance status (private, Medicaid, self-pay, “other” [“any other type of health insurance or health coverage plan”]). For the purposes of these analyses, Medicare was considered private insurance, and self-pay was inclusive of those who were either uninsured or elected to pay “out of pocket.”

#### Posttraumatic Stress Disorder Checklist for DSM-5

The Posttraumatic Stress Disorder Checklist for DSM-5 (PCL-5) is a PTSD symptom survey with scores ranging 0–80. A score of 33 or higher was used to indicate probable PTSD, based upon cutoffs identified in prior research and used to evaluate PTSD in prior TBI studies.^3,9^

#### Patient Health Questionnaire-9

The Patient Health Questionnaire-9 (PHQ-9) is a nine-item self-report screening tool for depressive symptoms with scores ranging from 0 to 27.^10^ A score of 15 or higher has been shown to be indicative of moderately severe to severe major depressive disorder. This cutoff was used in the present study and has been used in prior research to measure depression after TBI.^3^

#### Brief Symptom Inventory-18

The Brief Symptom Inventory-18 (BSI-18)^11^ is an 18-item self-report tool for psychological symptoms within the domains of somatization, depression, and anxiety. Raw scores are normalized to T scores, which range from 0 to 100. The T scores on the anxiety subscale were used in the present analysis, with ≥63 indicative of clinically significant anxiety.

### Statistical analysis

Descriptive statistics were calculated for each demographic, medical history, and injury variable for the overall sample and for groups with and without a preinjury psychiatric history. The analyzed cohort was stratified by history of psychiatric disorder (yes/no) to calculate rates of probable PTSD, MDD, or ANX from year 1 to year 7 post-injury. Complete-case analysis was used for all models. Due to variable follow-up rates, the rates of these disorders were also reported for years 2–4 and 5–7 post-injury. If multiple responses from a single participant were recorded in the 2–4 and 5–7 years range, only the first instance was utilized to not include duplicate responses. Univariable logistic regressions were built to evaluate the relationship between demographic and clinical predictors of psychiatric sequelae with meeting clinical cutoffs for PTSD, MDD, and ANX at 1 year, 2–4 years, and 5–7 years post-injury. Multivariable, forward selection logistic regression models were built to predict meeting cutoffs for PTSD, MDD, and ANX at 1 year, 2–4 years, and 5–7 years post-injury. The cutoff for entry in the model was *p* ≤ 0.05 and *p* > 0.10 for exclusion from the model. If a covariate had a *p* value between those two thresholds, it remained in the final model. Candidate predictors in the model were psychiatric history, migraine history, TBI history, initial CT status, LOC, PTA, GCS, insurance type, highest level of care, mechanism of injury, years of education, age, female sex, race, and ethnicity. Adjusted odds ratios (aOR) and 95% CIs were reported for all variables included in the final model. Inverse probability weighting was used in all models to account for missing outcomes. Standardized mean differences are reported in Supplementary Table S1 for the differences in weights at each timepoint. Statistical significance was set to *p* < 0.05.

## Results

Cohort demographics can be viewed in Table 1, and rates of follow-up can be viewed in Supplementary Table S1. Rate of follow-up was 66% at 1-year, 30% at 2–4 years (*n* = 593), and 31% at 5–7 years (*n* = 605). Median follow-up time for the 2–4 year window was 3 years, and for the 5–7 year window was 5 years. Thirteen percent (*n* = 253) completed follow-up at all timepoints. The sample was on average 42.2 ± 17.7 years old, 34% female, 78% white, and 21% Hispanic. The percent of females among those with a psychiatric history was 20% higher than among those without. The percent of participants with a psychiatric history and history of migraines was 8% higher than those without a psychiatric history. No notable differences were observed between psychiatric history groups for age, years of education, injury cause, highest level of care, GCS, or head injury clinical signs.

**Table 1.** Sample Characteristics

	Total			Imbalance of follow-up					
		Psych history		1 yr SMD		2–4 yr SMD		5–7 yr SMD	
		No	Yes	Unwt	wt	Unwt	wt	Unwt	wt
									
Subjects	1,948	1,507	441						
Psych history									
No	1,507 (77%)			−0.02	0.02	−0.06	−0.05	−0.04	−0.01
Yes	441 (23%)			0.02	−0.02	0.06	0.05	0.04	0.01
Age									
Mean (SD)	42.2 (17.7)	41.9 (17.9)	43.2 (17.1)	0.04	−0.03	−0.02	−0.03	−0.03	−0.04
Sex									
Male	1,293 (66%)	1,068 (71%)	225 (51%)	−0.13	−0.07	−0.09	−0.07	−0.13	−0.06
Female	655 (34%)	439 (29%)	216 (49%)	0.13	0.07	0.09	0.07	0.13	0.06
Race									
A-White	1,500 (78%)	1,130 (76%)	370 (84%)	−0.03	−0.02	0.17	0.07	0.16	0.08
B-Black	321 (17%)	271 (18%)	50 (11%)	0.04	0.03	−0.15	−0.05	−0.14	−0.05
C-Asian	66 (3%)	59 (4%)	7 (2%)	0.04	−0.01	0.00	−0.01	−0.10	−0.09
D-Other	44 (2%)	31 (2%)	13 (3%)	0.06	0.03	−0.04	−0.03	0.05	0.03
Unknown	17	16	1	−0.39	−0.12	−0.25	−0.22	−0.14	−0.08
Hispanic									
No	1,536 (79%)	1,145 (77%)	391 (89%)	0.33	0.08	0.08	0.03	0.19	0.09
Yes	397 (21%)	349 (23%)	48 (11%)	−0.30	−0.07	−0.06	−0.02	−0.17	−0.07
Unknown	15	13	2	−0.24	−0.07	−0.21	−0.14	−0.22	−0.21
Education years									
Mean (SD)	13.5 (2.9)	13.4 (2.9)	13.7 (2.8)	0.41	0.09	0.25	0.10	0.34	0.13
Unknown	66	58	8	−0.65	−0.12	−0.22	−0.09	−0.35	−0.09
Injury cause									
A) MVC occupant	623 (32%)	476 (32%)	147 (33%)	−0.06	−0.02	−0.09	−0.03	−0.04	−0.01
B) MCC	160 (8%)	129 (9%)	31 (7%)	−0.08	−0.05	−0.12	−0.09	−0.06	−0.03
C) MVC (cyclist or pedestrian)	299 (15%)	234 (16%)	65 (15%)	0.15	0.08	0.09	0.03	0.07	0.01
D) Fall	529 (27%)	393 (26%)	136 (31%)	0.01	−0.03	0.04	0.02	0.05	0.02
E) Assault	123 (6%)	98 (7%)	25 (6%)	−0.13	−0.04	−0.08	−0.02	−0.12	−0.04
F) Other/unknown	214 (11%)	177 (12%)	37 (8%)	0.05	0.05	0.11	0.06	0.04	0.01
Patient type									
1—ED only	493 (25%)	379 (25%)	114 (26%)	0.13	0.08	−0.24	−0.08	0.21	0.07
2—Hospital admit	841 (43%)	635 (42%)	206 (47%)	0.06	0.01	0.15	0.06	−0.09	−0.04
3—ICU admit	614 (32%)	493 (33%)	121 (27%)	−0.19	−0.08	0.04	0.01	−0.12	−0.02
ER GCS									
Mean (SD)	14.7 (0.5)	14.7 (0.5)	14.7 (0.6)	0.00	−0.02	−0.05	−0.03	0.13	0.07
13–14	458 (24%)	347 (23%)	111 (25%)	0.00	0.02	0.06	0.05	−0.13	−0.07
15	1,490 (76%)	1,160 (77%)	330 (75%)	0.00	−0.02	−0.06	−0.05	0.13	0.07
Insurance status									
A—Private	1,226 (65%)	933 (64%)	293 (68%)	0.26	0.03	0.22	0.10	0.22	0.07
B—Medicaid	214 (11%)	151 (10%)	63 (15%)	−0.07	−0.01	−0.09	−0.04	−0.07	−0.01
C—Self pay	389 (21%)	319 (22%)	70 (16%)	−0.14	−0.02	−0.18	−0.08	−0.12	−0.02
D—Other	58 (3%)	52 (4%)	6 (1%)	0.05	0.05	0.06	0.04	0.01	0.02
Unknown	61	52	9	−0.53	−0.11	−0.16	−0.08	−0.44	−0.25
Initial CT									
Negative	1,191 (63%)	908 (62%)	283 (66%)	0.05	0.04	−0.14	−0.06	0.14	0.07
Positive	697 (37%)	548 (38%)	149 (34%)	0.01	−0.01	0.12	0.04	−0.09	−0.04
Unknown	60	51	9	−0.20	−0.07	0.05	0.04	−0.16	−0.11
TBI history									
None	1,424 (78%)	1,146 (81%)	278 (69%)	0.14	0.06	0.05	0.03	0.01	0.03
ED only	243 (13%)	165 (12%)	78 (19%)	−0.02	−0.01	−0.08	−0.06	0.00	0.00
Hospital admission	154 (8%)	106 (7%)	48 (12%)	−0.06	−0.05	0.03	0.02	−0.08	−0.04
Unknown	127	90	37	−0.16	−0.05	−0.02	−0.01	0.06	0.00
History of migraines									
No	1,838 (94%)	1,452 (96%)	386 (88%)	−0.14	−0.07	−0.07	−0.05	−0.09	−0.05
Yes	110 (6%)	55 (4%)	55 (12%)	0.14	0.07	0.07	0.05	0.09	0.05
LOC									
No	257 (14%)	202 (14%)	55 (13%)	0.01	0.00	−0.15	−0.09	0.09	0.04
Yes	1,595 (86%)	1,233 (86%)	362 (87%)	−0.02	−0.03	0.13	0.08	−0.06	−0.02
Unknown	96	72	24	0.02	0.04	0.00	0.00	−0.05	−0.04
PTA									
No	310 (18%)	246 (18%)	64 (16%)	0.00	0.00	−0.14	−0.10	0.09	0.05
Yes	1,458 (82%)	1,111 (82%)	347 (84%)	0.00	−0.01	0.14	0.09	−0.08	−0.03
Unknown	180	150	30	0.01	0.02	−0.04	−0.01	0.01	−0.03
Day 1 GFAP									
Median (IQR)	289 (70–867)	305 (80–914)	237 (39–713)	−0.05	−0.01	0.02	−0.03	−0.04	−0.02
Unknown	97	86	11	−0.26	−0.05	−0.16	−0.08	−0.14	−0.05
Day 1 UCHL-1									
Median (IQR)	172 (92–330)	179 (95–347)	160 (85–269)	−0.10	−0.03	−0.06	−0.01	−0.06	−0.05
Unknown	97	86	11	−0.26	−0.05	−0.16	−0.08	−0.14	−0.05
Litigation status at 12 mo									
Did/will not litigate	1,007 (79%)	779 (79%)	228 (76%)	1.85		0.40		0.52	
Did/will litigate	272 (21%)	201 (21%)	71 (24%)	0.51		0.21		0.21	
Unknown	669	527	142	−10.1		−0.77		−0.99	

ED, emergency department; GCS, Glasgow Coma Scale; SD, standard deviation; TBI, traumatic brain injury.

Prevalence at 1, 2–4, and 5–7 years post-TBI of probable PTSD, MDD, and ANX can be viewed in Table 2. Participants with a history of pre-injury psychiatric disorder met or exceeded clinical cutoffs for these disorders at approximately double the rate of participants without a history of psychiatric disorder at year 1 (16–28% vs. 5–13%), between years 2 and 4 (15–35% vs. 6–18%), and between years 5 and 7 (10–28% vs. 5–12%). Regardless of pre-injury history, meeting cutoffs for PTSD was the most common, followed by ANX and MDD.

**Table 2.** Outcome

Post-injury year	PCL5 ≥ 33		PHQ9 ≥ 15		BSI18 anxiety *T* ≥ 63	
	No psych Hx	Psych Hx	No psych Hx	Psych Hx	No psych Hx	Psych Hx
**1**	126/968 (13%)	82/291 (28%)	50/986 (5%)	47/293 (16%)	97/985 (10%)	69/294 (23%)
**2**	30/137 (22%)	19/63 (30%)	10/138 (7%)	9/67 (13%)	22/138 (16%)	19/67 (28%)
**3**	47/245 (19%)	29/86 (34%)	13/250 (5%)	10/91 (11%)	37/250 (15%)	20/91 (22%)
**4**	56/329 (17%)	38/104 (37%)	21/336 (6%)	21/107 (20%)	40/335 (12%)	29/107 (27%)
**5**	42/380 (11%)	34/115 (30%)	18/401 (4%)	12/117 (10%)	33/401 (8%)	22/117 (19%)
**6**	22/232 (9%)	16/80 (20%)	8/243 (3%)	6/82 (7%)	20/242 (8%)	14/82 (17%)
**7**	17/122 (14%)	6/40 (15%)	4/127 (3%)	5/41 (12%)	10/127 (8%)	4/41 (10%)
**8**	4/30 (13%)	1/10 (10%)	3/29 (10%)	1/10 (10%)	5/30 (17%)	1/10 (10%)
**9**	1/7 (14%)	0/3 (0%)	1/8 (13%)	0/3 (0%)	1/8 (13%)	0/4 (0%)
**10**	0/1 (0%)	—	0/1 (0%)	—	0/1 (0%)	—
**2–4**	80/444 (18%)	50/143 (35%)	25/447 (6%)	22/145 (15%)	57/446 (13%)	39/145 (27%)
**5–7**	55/444 (12%)	39/141 (28%)	22/461 (5%)	15/143 (10%)	39/460 (8%)	27/143 (19%)

BSI18, brief symptom inventory-18; PCL5, posttraumatic stress disorder checklist for DSM-5; PHQ9, patient health questionnaire-9.

### Regression modeling with the whole cohort

Regression modeling to predict meeting or exceeding clinical cutoffs for PTSD, MDD, and ANX at 1, 2–4, and 5–7 years post-injury can be viewed in Tables 3–5, respectively. In multivariable modeling at 1 year post-injury, psychiatric history (PTSD: aOR = 2.60, 95% CI: 1.81–3.73, MDD: aOR = 3.44, 95% CI: 2.18–5.44, ANX: aOR = 2.58, 95% CI: 1.75–3.81), Black race (vs. white; PTSD: aOR = 2.37, 95% CI: 1.44–3.90, MDD: aOR = 3.22, 95% CI: 1.41–7.36, ANX: aOR = 2.01, 95% CI: 1.14–3.54), and years of education (aOR = PTSD: aOR = 0.50, 95% CI: 0.39–0.64, MDD: aOR = 0.64, 95% CI: 0.45–0.89, ANX: aOR = 0.52, 95% CI: 0.40–0.69 per 4 years of education) were associated with meeting clinical cutoffs for each of the 3 disorders. Age (aOR = 0.88, 95% CI: 0.79–0.97 per 10 years), TBI history with ED visit (aOR = 1.45; 95% CI: 1.08–1.98), assault mechanism (aOR = 2.07; 95% CI: 1.36–3.17), and MVC (aOR = 0.68, 95% CI: 0.52–0.88) were additional predictors of PTSD. History of migraines was an additional predictor of MDD (aOR = 2.31; 95% CI: 1.16–4.60). Assault mechanism (aOR = 2.11; 95% CI: 1.33–3.36), MVC mechanism (aOR = 0.69; 95% CI: 0.52–0.92), prior TBI with hospital admission (aOR = 1.47; 95% CI: 1.01–2.16), and post-traumatic amnesia (aOR = 1.78; 95% CI: 1.06–3.00) were additional statistically significant predictors of ANX.

**Table 3.** Modeling 1-Year Outcome

	Predicting PCL5 ≥ 33				Predicting PHQ9 ≥ 15				Predicting BSI-Anx *T* ≥ 63			
	Univariable		Multivariable forward selection		Univariable		Multivariable forward selection		Univariable		Multivariable forward selection	
	OR	*p*	OR	*p*	OR	*p*	OR	*p*	OR	*p*	OR	*P*
Psych history	2.52 (1.84, 3.45)	<0.001	2.60 (1.81, 3.73)	<0.001	3.46 (2.27, 5.27)	<0.001	3.44 (2.18, 5.44)	<0.001	2.71 (1.93, 3.81)	<0.001	2.58 (1.75, 3.81)	<0.001
Age (per +10yrs)	0.86 (0.79, 0.94)	0.001	0.88 (0.79, 0.97)	0.013	0.98 (0.87, 1.10)	0.725			0.94 (0.86, 1.03)	0.201		
Female	1.37 (1.01, 1.85)	0.041	1.41 (1.00, 1.99)	0.051	1.51 (0.99, 2.29)	0.053			1.02 (0.73, 1.42)	0.920		
Race (vs White)	—	<0.001	—	<0.001	—	<0.001	—	<0.001	—	0.002	—	0.010
Black	2.38 (1.69, 3.35)	<0.001	2.37 (1.44, 3.90)	<0.001	2.79 (1.78, 4.36)	<0.001	3.22 (1.41, 7.36)	0.005	1.89 (1.30, 2.76)	0.001	2.01 (1.14, 3.54)	0.016
Asian	0.37 (0.10, 1.33)	0.126	0.72 (0.26, 2.01)	0.531	0.34 (0.04, 2.66)	0.305	0.75 (0.14, 3.90)	0.729	0.51 (0.15, 1.73)	0.277	1.10 (0.40, 3.03)	0.859
Other/unknown	0.65 (0.22, 1.93)	0.434	0.57 (0.23, 1.43)	0.233	0.33 (0.03, 3.20)	0.341	0.39 (0.07, 2.28)	0.293	0.40 (0.09, 1.75)	0.222	0.45 (0.14, 1.47)	0.186
Hispanic	1.27 (0.89, 1.81)	0.190			0.68 (0.38, 1.21)	0.186			0.97 (0.64, 1.46)	0.878		
Educ. years (per +4yrs)	0.51 (0.41, 0.64)	<0.001	0.50 (0.39, 0.64)	<0.001	0.64 (0.48, 0.85)	0.002	0.64 (0.45, 0.89)	0.009	0.55 (0.44, 0.70)	<0.001	0.52 (0.40, 0.69)	<0.001
Cause (vs Fall)	—	<0.001	—	0.002	—	0.674			—	<0.001	—	0.006
Assault	3.28 (1.88, 5.74)	<0.001	2.07 (1.36, 3.17)	0.001	0.99 (0.38, 2.55)	0.978			3.65 (2.03, 6.57)	<0.001	2.11 (1.33, 3.36)	0.002
MVC	1.20 (0.83, 1.72)	0.326	0.68 (0.52, 0.88)	0.004	1.14 (0.71, 1.84)	0.592			1.22 (0.82, 1.82)	0.324	0.69 (0.52, 0.92)	0.012
Other/unknown	1.28 (0.75, 2.19)	0.359	0.89 (0.59, 1.33)	0.558	0.71 (0.31, 1.63)	.421			1.10 (0.60, 2.01)	0.765	0.89 (0.57, 1.39)	0.610
Pt.type (vs ED dischg)	—	0.629			—	0.779			—	0.419		
Inpatient no ICU	0.87 (0.59, 1.27)	0.468			1.17 (0.67, 2.06)	0.578			1.25 (0.80, 1.95)	0.328		
ICU	0.84 (0.59, 1.21)	0.352			1.20 (0.71, 2.04)	.493			1.32 (0.87, 1.99)	0.192		
GCS 15	1.30 (0.90, 1.87)	0.157			1.71 (0.98, 2.98)	0.060			1.18 (0.80, 1.74)	0.413		
Insurance (vs private)	—	<0.001			—	0.001			—	0.001		
Medicaid	2.53 (1.66, 3.86)	<0.001			2.80 (1.64, 4.79)	<0.001			2.30 (1.46, 3.64)	<0.001		
Self-pay	1.98 (1.38, 2.83)	<0.001			1.37 (0.81, 2.32)	0.236			1.67 (1.12, 2.48)	0.011		
Other/unknown	1.28 (0.63, 2.58)	0.498			0.51 (0.13, 1.98)	0.331			1.23 (0.58, 2.62)	0.591		
CT positive	0.76 (0.55, 1.04)	0.090			0.75 (0.48, 1.18)	0.218			1.12 (0.80, 1.56)	0.515		
TBI history (vs none)	—	<0.001	—	<0.001	—	<0.001	—	0.001	—	<0.001	—	<0.001
ED visit only	2.56 (1.74, 3.77)	<0.001	1.46 (1.08, 1.98	0.014	2.60 (1.55, 4.37)	<0.001	1.27 (0.86, 1.86)	0.225	2.21 (1.44, 3.39)	<0.001	1.20 (0.85, 1.68)	0.300
Hospital admission	2.08 (1.28, 3.39)	0.003	1.22 (0.86, 1.73)	0.274	2.73 (1.47, 5.06)	0.001	1.41 (0.91, 2.17)	0.121	2.47 (1.49, 4.11)	<0.001	1.47 (1.01, 2.16)	0.045
Hx of migraines	1.88 (1.11, 3.20)	0.020			2.75 (1.47, 5.15)	0.002	2.31 (1.16, 4.60)	0.018	2.16 (1.25, 3.72)	0.006	1.83 (0.96, 3.51)	0.067
LOC	1.43 (0.89, 2.31)	0.144			1.50 (0.75, 3.00)	0.256			1.54 (0.90, 2.64)	0.113		
PTA	1.31 (0.86, 2.00)	0.210			1.22 (0.67, 2.19)	0.516			1.31 (0.82, 2.09)	0.252	1.78 (1.06, 3.00)	0.030
*N* due to missing outcomes			1,259 (65%)				1,279 (66%)				1,279 (66%)	
*N* due to missing covariates			1,181 (94%)				1,198 (94%)				1,095 (86%)	
Nagelkerke *R*^2^			18.5				15.2%				15.3%	
Concordance			0.75				0.75				0.73	

All predictors assessed with univariable models were included as potential predictors in the forward selection, multivariable models.

All analyses using propensity-weighting to account for missing outcomes.

BSI, brief symptom inventory; ED, emergency department; GCS, Glasgow Coma Scale; OR, odds ratio; PCL5, posttraumatic stress disorder checklist for DSM-5; PHQ9, patient health questionnaire-9; TBI, traumatic brain injury.

**Table 4.** Modeling 2–4 Year Outcome

	Predicting PCL5 ≥ 33				Predicting PHQ9 ≥ 15				Predicting BSI-Anx *T* ≥ 63			
	Univariable		Multivariable forward selection		Univariable		Multivariable forward selection		Univariable		Multivariable forward selection	
	OR	*p*	OR	*p*	OR	*p*	OR	*p*	OR	*p*	OR	*p*
Psych history	2.70 (1.78, 4.09)	<0.001	2.46 (1.51, 4.01)	0.001	3.00 (1.69, 5.35)	<0.001	3.34 (1.58, 7.10)	0.002	2.55 (1.62, 4.00)	<0.001	2.83 (1.67, 4.79)	<0.001
Age (per +10 yrs)	0.85 (0.76, 0.96)	0.007			0.93 (0.79, 1.10)	0.423			0.88 (0.78, 1.01)	0.060		
Female	1.47 (0.99, 2.17)	0.055			1.08 (0.60, 1.93)	0.800			0.72 (0.46, 1.14)	0.166	0.55 (0.32, 0.96)	0.034
Race (vs White)	—	0.001	—	.002	—	0.002	—	0.016	—	0.054		
Black	1.81 (1.10, 2.96)	0.018	2.13 (1.22, 3.72)	.008	3.22 (1.71, 6.05)	<0.001	3.60 (1.58, 5.21)	0.002	2.07 (1.23, 3.49)	0.006		
Asian	—	—	—	—	—	—	—	—	—	—		
Other/unknown	7.80 (2.38, 25.57)	0.001	7.07 (1.97, 25.4)	0.003	3.78 (0.97, 14.74)	0.056	3.31 (0.67, 16.4)	0.142	1.50 (0.39, 5.72)	0.552		
Hispanic	0.75 (0.45, 1.25)	0.276			0.94 (0.46, 1.94)	0.877			1.02 (0.60, 1.74)	0.942		
Educ. years (per +4yrs)	0.48 (0.36, 0.65)	<0.001	0.45 (0.32, 0.62)	<0.001	0.53 (0.36, 0.79)	0.002			0.45 (0.33, 0.63)	<0.001	0.47 (0.32, 0.68)	<0.001
Cause (vs fall)	—	0.515			—	0.107			—	0.018		
Assault	1.36 (0.61, 3.06)	0.454			2.55 (0.96, 6.74)	0.060			3.69 (1.64, 8.34)	0.002		
MVC	0.92 (0.59, 1.45)	0.725			0.80 (0.41, 1.58)	0.528			1.36 (0.80, 2.32)	0.256		
Other/unknown	1.35 (0.72, 2.53)	0.354			1.13 (0.44, 2.88)	0.798			1.30 (0.61, 2.80)	0.498		
Pt.type (vs ED dischg)	—	0.112			—	0.080			—	0.016		
Inpatient no ICU	1.45 (0.83, 2.54)	0.197			2.77 (1.12, 6.84)	0.027			2.69 (1.35, 5.35)	0.005		
ICU	1.75 (1.03, 2.96)	0.037			1.93 (0.78, 4.74)	0.153			2.38 (1.22, 4.62)	0.011		
GCS 15	1.10 (0.70, 1.72)	0.680			0.65 (0.36, 1.19)	0.163			0.67 (0.42, 1.06)	0.089		
Insurance (vs private)	—	<0.001			—	<0.001	—	0.003	—	<0.001	—	<0.001
Medicaid	3.78 (2.14, 6.67)	<0.001			5.02 (2.31, 10.92)	<0.001	1.84 (0.86, 3.95)	0.119	5.04 (2.75, 9.25)	<0.001	2.27 (1.32, 3.92)	0.003
Self-pay	1.27 (0.75, 2.13)	0.370			3.38 (1.67, 6.85)	0.001	1.39 (0.71, 2.73)	0.334	2.49 (1.44, 4.29)	0.001	1.14 (0.70, 1.86)	0.609
Other/unknown	1.00 (0.41, 2.43)	0.998			4.01 (1.46, 10.97)	0.007	1.02 (0.30, 3.55)	0.969	2.59 (1.12, 6.01)	0.027	0.69 (0.31, 1.58)	0.385
CT positive	0.64 (0.42, 0.96)	0.032			1.40 (0.78, 2.50)	0.255	1.99 (0.93, 4.22)	0.074	0.90 (0.57, 1.42)	0.661		
TBI history (vs none)	—	<0.001	—	0.003	—	0.019	—	0.035	—	0.127		
ED visit only	2.05 (1.16, 3.61)	0.013	0.91 (0.58, 1.42)	0.676	0.81 (0.29, 2.27)	0.683	0.36 (0.15, 0.85)	0.021	1.37 (0.71, 2.63)	0.345		
Hospital admission	3.47 (1.89, 6.39)	<0.001	1.87 (1.18, 2.99)	0.008	2.92 (1.34, 6.37)	0.007	3.02 (1.47, 6.19)	0.003	1.96 (0.99, 3.89)	0.055		
Hx of migraines	3.50 (1.80, 6.81)	<0.001	5.00 (2.20, 11.40)	<0.001	2.04 (0.82, 5.10)	0.126			1.71 (0.80, 3.66)	0.165	2.21 (0.93, 5.22)	0.072
LOC	0.92 (0.50, 1.70)	0.799			0.68 (0.31, 1.52)	0.351	0.24 (0.08, 0.69)	0.008	1.36 (0.64, 2.88)	0.427		
PTA	0.94 (0.53, 1.67)	0.842			3.80 (1.01, 14.34)	0.048	4.68 (1.08, 20.27)	0.039	2.54 (1.12, 5.75)	0.025		
*N* due to missing outcomes			587 (30%)				592 (30%)				591 (30%)	
*N* due to missing covariates			543 (93%)				468 (79%)				505 (85%)	
Nagelkerke *R*^2^			24.3%				26.2%				19.2%	
Concordance			0.77				0.82				0.74	

All predictors assessed with univariable models were included as potential predictors in the forward selection, multivariable models.

All analyses using propensity-weighting to account for missing outcomes.

BSI, brief symptom inventory; ED, emergency department; GCS, Glasgow Coma Scale; OR, odds ratio; PCL5, posttraumatic stress disorder checklist for DSM-5; PHQ9, patient health questionnaire-9; TBI, traumatic brain injury.

**Table 5.** Modeling 5–7 Year Outcome

	Predicting PCL5 ≥ 33				Predicting PHQ9 ≥ 15				Predicting BSI-Anx *T* ≥ 63			
	Univariable		Multivariable forward selection		Univariable		Multivariable forward selection		Univariable		Multivariable forward selection	
	OR	*p*	OR	*p*	OR	*p*	OR	*p*	OR	*p*	OR	*p*
Psych history	2.58 (1.62, 4.10)	<0.001	3.75 (2.13, 6.62)	<0.001	2.23 (1.14, 4.36)	0.019			2.74 (1.64, 4.59)	<0.001	2.84 (1.65, 4.88)	<0.001
Age (per +10yrs)	0.96 (0.84, 1.09)	0.492			0.90 (0.74, 1.10)	0.315			0.84 (0.72, 0.98)	0.026	0.82 (0.70, 0.97)	0.022
Female	2.44 (1.57, 3.80)	<0.001	2.45 (1.46, 4.11)	0.001	1.88 (0.98, 3.58)	0.056			0.99 (0.59, 1.65)	0.957		
Race (vs White)	—	0.001	—	0.002	—	0.909			—	0.181		
Black	2.71 (1.60, 4.57)	<0.001	2.43 (1.10, 5.37)	0.029	1.36 (0.60, 3.09)	0.466			1.29 (0.67, 2.50)	0.450		
Asian	0.33 (0.03, 3.92)	0.381	0.37 (0.05, 2.51)	0.308	—	—			—	—		
Other/unknown	2.49 (0.86, 7.17)	0.091	1.51 (0.52, 4.40)	0.446	0.95 (0.13, 6.79)	0.962			3.20 (1.11, 9.25)	0.032		
Hispanic	0.67 (0.36, 1.26)	0.216			1.42 (0.66, 3.04)	0.369			0.80 (0.41, 1.57)	0.520		
Educ. years (per +4yrs)	0.62 (0.44, 0.87)	0.006			0.68 (0.41, 1.11)	0.123			0.62 (0.42, 0.91)	0.016		
Cause (vs fall)	—	0.098			—	0.669			—	0.430		
Assault	2.44 (0.96, 6.23)	0.061			1.49 (0.34, 6.62)	0.597			2.02 (0.73, 5.59)	0.177		
MVC	1.71 (0.96, 3.02)	0.067			1.46 (0.64, 3.32)	0.367			1.24 (0.67, 2.29)	0.490		
Other/unknown	2.32 (1.09, 4.95)	0.029			1.96 (0.66, 5.83)	0.225			1.73 (0.75, 3.99)	0.197		
Pt.type (vs ED dischg)	—	0.173			—	0.304			—	0.103		
Inpatient no ICU	0.89 (0.48, 1.65)	0.714			0.73 (0.29, 1.87)	0.514			0.68 (0.33, 1.40)	0.297		
ICU	1.42 (0.83, 2.43)	0.204			1.37 (0.63, 2.98)	0.422			1.35 (0.75, 2.43)	0.322		
GCS 15	1.65 (0.91, 3.00)	0.100	1.79 (0.89, 3.63)	0.104	1.34 (0.57, 3.14)	0.496			1.70 (0.85, 3.38)	0.131		
Insurance (vs private)	—	0.001	—	0.003	—	0.005	—	0.006	—	0.014		
Medicaid	3.00 (1.62, 5.59)	0.001	1.04 (0.58, 1.86)	0.891	4.32 (1.90, 9.87)	0.001	2.12 (1.08, 4.14)	0.029	2.82 (1.43, 5.58)	0.003		
Self-pay	2.01 (1.17, 3.44)	0.011	1.51 (0.91, 2.51)	0.109	2.25 (1.01, 5.00)	0.047	1.22 (0.64, 2.32)	0.545	1.85 (1.01, 3.38)	0.046		
Other/unknown	2.13 (0.78, 5.84)	0.141	1.26 (0.56, 2.85)	0.571	1.86 (0.40, 8.64)	0.429	0.83 (0.27, 2.56)	0.745	0.98 (0.25, 3.93)	0.982		
CT positive	0.66 (0.41, 1.07)	0.090			0.68 (0.32, 1.43)	0.311			0.85 (0.50, 1.45)	0.540		
TBI history (vs none)	—	0.003			—	0.581			—	0.019		
ED visit only	2.23 (1.24, 4.02)	0.008			1.57 (0.67, 3.68)	0.299			1.88 (0.96, 3.67)	0.064		
Hospital admission	2.58 (1.27, 5.24)	0.009			1.15 (0.34, 3.87)	0.823			2.62 (1.20, 5.69)	0.015		
Hx of migraines	2.75 (1.35, 5.60)	0.005			3.31 (1.34, 8.18)	0.010			3.31 (1.57, 6.97)	0.002	2.58 (1.16, 5.75)	0.020
LOC	1.49 (0.74, 3.00)	0.268	2.27 (1.00, 5.19)	0.051	3.97 (0.81, 19.44)	0.089	3.75 (0.75, 18.60)	0.106	1.71 (0.74, 3.99)	0.211		
PTA	0.62 (0.36, 1.06)	0.081			1.24 (0.49, 3.10)	0.651			1.56 (0.73, 3.32)	0.247		
*N* due to missing outcomes			585 (30%)				604 (31%)				603 (31%)	
*N* due to missing covariates			519 (89%)				537 (89%)				558 (93%)	
Nagelkerke *R*^2^			21.1%				7.6%				8.5%	
Concordance			0.77				0.69				0.67	

All predictors assessed with univariable models were included as potential predictors in the forward selection, multivariable models.

All analyses using propensity-weighting to account for missing outcomes.

BSI, brief symptom inventory; ED, emergency department; GCS, Glasgow Coma Scale; OR, odds ratio; PCL5, posttraumatic stress disorder checklist for DSM-5; PHQ9, patient health questionnaire-9; TBI, traumatic brain injury

At 2–4 years post-injury, psychiatric history (PTSD: aOR = 2.46, 95% CI: 1.51–4.01; MDD: aOR = 3.34, 95% CI: 1.58–7.10; ANX: aOR = 2.83, 95% CI: 1.67–4.79) was associated with meeting clinical cutoffs for all 3 disorders. Black race (aOR = 2.13; 95% CI: 1.22–0.72), other/unknown race (aOR = 7.07; 95% CI: 1.97–25.40), years of education (aOR = 0.45; 95% CI: 0.32–0.62), prior TBI history with hospital admission (aOR = 1.87; 95% CI: 1.18–2.99), and history of migraines (aOR = 5.00; 95% CI: 2.20–11.40) were additional predictors of PTSD. Black race (aOR = 3.60; 95% CI: 1.58–5.21), prior TBI with hospital admission (aOR = 3.02; 95% CI: 1.47–6.19), TBI history with ED visit only (aOR = 0.36; 95% CI: 0.15–0.85), LOC (aOR = 0.24; 95% CI: 0.08–0.69), and PTA (aOR = 4.68; 95% CI: 1.08–20.27) were additional predictors of MDD. Female sex (aOR = 0.55; 95% CI: 0.32–0.96), years of education (aOR = 0.47; 95% CI: 0.32–0.68), and Medicaid insurance (aOR = 2.27; 95% CI: 1.32–3.92) were additional statistically significant predictors of ANX.

At 5–7 years post-injury, psychiatric history (PTSD: aOR = 3.75; 95% CI: 2.13–6.62). Female sex (aOR = 2.45; 95% CI: 1.46–4.11) and Black race (aOR = 2.43; 95% CI: 1.10–5.37) were predictors of PTSD. Medicaid insurance was the only statistically significant predictor of MDD (aOR = 2.12; 95% CI: 1.08–4.14). Psychiatric history (aOR = 2.84; 95% CI: 1.65–4.88), age (aOR = 0.82; 95% CI: 0.70–0.97 per 10 years), and history of migraines (aOR = 2.58; 95% CI: 1.16–5.75) were significant predictors of ANX.

## Discussion

The results of this study suggest that history of psychiatric disorder (obtained in this study by self-report) is the most robust predictor of post-TBI prevalence for meeting clinical cutoffs for PTSD, MDD, and/or ANX included in this study. De novo psychiatric conditions were still common in participants who did not report a psychiatric disorder history prior to TBI, with prevalence rates between 9% and 22% for PTSD, 3% and 7% for MDD, and 8% and 16% for ANX at 1, 2–4, and 5–7 years post-injury. Multivariable modeling confirmed the importance of several classical risk factors for post-TBI psychiatric sequelae, such as pre-injury psychiatric history, prior TBI history, and Black race. Identified risk factors largely mirror prior findings for predicting 6-month outcomes in this population,^3,12^ suggesting these factors are robust indicators of long-term psychiatric issues. Type of insurance (Medicare or self-pay vs. private) was associated with meeting cutoffs for each of the three disorders at 1 -year, 2–4 years, and 5–7 years post-injury, indicating a potential long-term impact of insurance type on post-traumatic psychiatric health (although insurance type could also be a proxy for socioeconomic status). The present study indicates that, for many patients in this population, psychiatric symptoms are common up to 7 years post-TBI and can be magnified by pre-injury health conditions and individual life experiences.

Prevalence for PTSD fluctuates widely across studies due to variable criteria and study populations. A recent systematic review identified the lifetime prevalence of PTSD among US civilians between 3.4% and 26.9%, with a one-year prevalence from 2.3% to 9.1%.^13^ Rates are substantially higher among military/veteran populations whose exposure to traumatic events occurs at a much higher frequency than civilians.^14^ Within this civilian sample, assault mechanism of injury was associated with ∼2.5 times higher odds of meeting cutoffs for PTSD and anxiety at 1 year post-injury.^3^ Assault cause of injury, especially in the context of intimate partner violence,^15–17^ is a growing focus of research in the TBI literature as a risk factor for worse outcomes.^12^ This is a critical area of future research, as assault is a well-characterized risk factor for PTSD after trauma of any kind.^18,19^ Context of injury should be considered more frequently as a predictor of post-TBI psychiatric issues and in future studies.^16^

Based upon the DSM-5 definition, the national prevalence of major depressive disorder in US adults for the prior 12 months is 10.4%, with a lifetime prevalence of 20.6%.^20^ Participants of the present study who did not report prior psychiatric disorder had lower rates of meeting study criteria for major depressive disorder than the national average (3–7%). Conversely, participants with psychiatric disorder exceeded the national average from 1 to 4 years post-injury (15–16%) before normalizing in years 5–7. These results demonstrate the importance of pre-injury psychiatric health on post-injury psychiatric symptoms. Regardless of pre-injury status, depression is a potential long-term consequence of TBI. A recent systematic review including >106,000 patients with TBI and GCS 13–15 concluded that a TBI history within the prior 10 years was associated with three times increased odds of depression compared with those without prior TBI history.^21^ Developing evidence-based treatment protocols for TBI-associated depression constitutes an increasingly relevant clinical need and includes candidates such as transcranial magnetic stimulation,^22^ pharmacotherapies,^23^ and light therapies.^24^ TBI-associated depression may be a distinct physiological disorder from other psychiatric conditions whose genesis occurred independent of TBI.^25^

According to the U.S. Centers for Disease Control and Prevention, 15.6% of adults in the US experience at least mild anxiety, which coincides with the post-TBI prevalence identified in this study for those with no psychiatric history (8–16%).^26^ A recent systematic review of 20 studies including only *de novo* anxiety cases following TBI found that patients were more prone to generalized ANX compared with other anxiety conditions.^27^ In the present study, participants with history of a psychiatric disorder had markedly higher rates of meeting the clinical cutoff for anxiety at 1 year (23%), 2–4 years (27%), and 5–7 years post-injury (19%) than participants without history of a psychiatric disorder. Future work should evaluate how sociocultural predictors may be associated with psychiatric sequelae after TBI and provide targeted follow-up care to individuals at risk for developing psychiatric sequelae.

Timing of and adherence to therapies for TBI symptoms were unavailable for the present study. It is important to note that early initiation of targeted therapies may improve outcomes for all TBI patients but may be especially important for those experiencing post-injury psychiatric symptoms.^28–30^ The present study demonstrates that meeting clinical cutoffs for psychiatric conditions following TBI can persist for years, underscoring the importance of early screening and intervention in this population. Seabury et al.^31^ previously reported that nearly half of adults evaluated for GCS 13–15 TBI at a U.S. Level 1 trauma center ED do not report receiving educational material at discharge, which could reinforce the importance of early intervention to the patient. Furthermore, even in those patients experiencing clinically relevant long-term symptoms, over half of patients with TBI first seen in the ED do not follow up with a medical professional.^31,32^ Increasing education and encouragement to seek follow-up care is critically important for all patients with TBI but may be especially important for the patient with a pre-injury psychiatric history. Prior work has demonstrated that targeted therapies, such as cognitive behavioral therapy, actually have higher efficacy following TBI in patients with higher pre-injury depression or anxiety symptoms.^33^ Clinicians who treat patients with several of the risk factors reported in the current work should consider increasing outreach methods to ensure specialized follow-up care is sought.

### Limitations

While screening questionnaires with validated clinical cutoffs were used to determine probable PTSD, MDD, and ANX, meeting clinical cutoffs does not guarantee that a patient would be diagnosed with these disorders by a clinician. Prior work in this population has used different cutoffs for the outcome measures used in this study, such as a total score of 10 for PHQ-9 and a total score of 7 for GAD-7.^34^ Data collection procedures precluded estimating the degree to which the index injury leading to study enrollment contributed to reported psychiatric symptoms. Pre-injury medical history was self-reported by the participant, which increases risk for recall bias. It is possible that participants who did not report a pre-injury psychiatric disorder diagnosis actually had an undiagnosed psychiatric disorder prior to their TBI. Future research should consider the potential differential effect of certain psychiatric disorders on rates of post-TBI psychiatric issues. Loss to follow-up was observed in this study, which could have affected outcomes, but rates were consistent with prior longitudinal TBI research studies.^35,36^ Loss to follow-up was addressed in modeling but inverse probability weighting, but changes in sample size may have influenced the identified predictors at different timepoints. The results of this study are generalizable to adults presenting to U.S. Level 1 trauma centers with GCS 13–15 TBI.

## Conclusion

In this analysis of adults with GCS 13–15 TBI at U.S. Level 1 trauma centers, history of pre-injury psychiatric disorder was associated with about double the prevalence of meeting clinical cutoffs for PTSD, MDD, and ANX from 1 to 7 years post-injury. The results of this study indicate that probable PTSD, MDD, and ANX were more common in participants with pre-injury psychiatric disorder; however, psychiatric symptoms can occur for years post-injury, even among those with *de novo* psychiatric disorders. Psychiatric history constitutes an integral component of TBI assessment and long-term monitoring. Sociocultural influence on long-term psychiatric sequelae after TBI requires careful consideration in contemporary TBI studies.

## Supplementary Material

Supplementary Table S1

## Data Availability

De-identified data are available by request from the Federal Interagency TBI Research Informatics System.

## The TRACK-TBI Investigators

Ann-Christine Duhaime, MD, Harvard Medical School; Shankar Gopinath, MD, Baylor College of Medicine; Vijay Krishnamoorthy, MD, Duke University; Christine Mac Donald, PhD, University of Washington; Randall Merchant, PhD, Virginia Commonwealth University; Pratik Mukherjee, MD, PhD, University of California, San Francisco; Laura B. Ngwenya, MD, PhD, University of Cincinnati; Claudia Robertson, MD, Baylor College of Medicine; David Schnyer, PhD, Sabrina R. Taylor, PhD; University of California, San Francisco; Ross Zafonte, DO, Harvard Medical School

## Transparency, Rigor, and Reproducibility Statement

This is a secondary analysis of a prospective cohort study, so the study was not preregistered. The analysis plan for the current study was not preregistered online but was conceived by the primary authors and executed by a biostatistician. The primary author certifies that the analyses were prespecified. The sample size was a convenience sample of participants enrolled as part of a multisite prospective consortium. Data collection and analyses were performed by investigators who were aware of relevant characteristics. Data and analytic code are available at reasonable request. R was used to complete analyses.

## Abbreviations Used

ANX: Anxiety
aOR: Adjusted odds ratio
BSI-18: Brief Symptom Inventory 18
CT: Computed tomography
ED: Emergency department
GCS: Glasgow Coma Scale
GFAP: Glial Fibrillary Acidic Protein
LOC: Loss of consciousness
MDD: Major Depression Disorder
MCC: Motor cycle collision
PHQ9: Patient Health Questionnaire 9
PTA: Post-traumatic amnesia
PTSD: Post-Traumatic Stress Disorder
PCL-5: Post-traumatic stress disorder checklist for DSM-5
TBI: Traumatic brain injury
TRACK-TBI: Transforming Research and Clinical Knowledge in TBI
UCH-L1: Ubiquitin c-Terminal Hydrolase-L1
US: United States
