# Supplementary material for: Prior Psychiatric Disorder and Post-Traumatic Stress, Depressive and Anxiety Disorder after Traumatic Brain Injury with Glasgow Coma Scale Score 13–15: A TRACK-TBI Study
Source: Neurotrauma Rep. 2025 Sep 30;6(1):944–55. doi: 10.1177/2689288X251383348 (PMC12549169; doi:10.1177/2689288X251383348)
Supplement: Supplementary Table S1 [file 2689288x251383348_supplementary_table_s1.docx]

**Supplemental Table 1 -- Follow-Up Rates**

| **1 Year** | | **Overall** | | **PCL** | | **PHQ** | | **BSI** | |
| --- | --- | --- | --- | --- | --- | --- | --- | --- | --- |
|  | |  | |  | |  | |  | |
| **Followed** | |  | |  | |  | |  | |
| No | | 667 (34%) | | 689 (35%) | | 669 (34%) | | 669 (34%) | |
| Yes | | 1281 (66%) | | 1259 (65%) | | 1279 (66%) | | 1279 (66%) | |
|  | |  | |  | |  | |  | |
| **2-4 Year** | | **Overall** | | **PCL** | | **PHQ** | | **BSI** | |
|  | |  | |  | |  | |  | |
| **Followed** | |  | |  | |  | |  | |
| No | | 1355 (70%) | | 1361 (70%) | | 1356 (70%) | | 1357 (70%) | |
| Yes | | 593 (30%) | | 587 (30%) | | 592 (30%) | | 591 (30%) | |
|  | |  | |  | |  | |  | |
| **Follow-Up Times** | |  | |  | |  | |  | |
| Median (IQR) years | | 3 (2-4) | | 3 (2-4) | | 3 (2-4) | | 3 (2-4) | |
| 2 years | |  | | 200 (34%) | | 205 (35%) | | 205 (35%) | |
| 3 years | |  | | 189 (32%) | | 188 (32%) | | 188 (32%) | |
| 4 years | |  | | 198 (34%) | | 199 (34%) | | 198 (34%) | |
| Not Followed | |  | | 1361 | | 1356 | | 1357 | |
|  | |  | |  | |  | |  | |
| **5-7 Year** | | **Overall** | | **PCL** | | **PHQ** | | **BSI** | |
|  | |  | |  | |  | |  | |
| **Followed** | |  | |  | |  | |  | |
| No | | 1343 (69%) | | 1363 (70%) | | 1344 (69%) | | 1345 (69%) | |
| Yes | | 605 (31%) | | 585 (30%) | | 604 (31%) | | 603 (31%) | |
|  | |  | |  | |  | |  | |
| **Follow-Up Times** | |  | |  | |  | |  | |
| Median (IQR) years | | 5 (5-5) | | 5 (5-5) | | 5 (5-5) | | 5 (5-5) | |
| 5 years | |  | | 495 (85%) | | 518 (86%) | | 518 (86%) | |
| 6 years | |  | | 72 (12%) | | 69 (11%) | | 68 (11%) | |
| 7 years | |  | | 18 (3%) | | 17 (3%) | | 17 (3%) | |
| Not Followed | |  | | 1363 | | 1344 | | 1345 | |
